# Predictors for Dexmedetomidine Requirement for Sedation under Regional Anesthesia

**DOI:** 10.3390/jcm13051435

**Published:** 2024-03-01

**Authors:** Jun Ho Lee, Taehyeon Jung, Seonghoon Ko, Aram Doo

**Affiliations:** 1 Department of Anesthesiology and Pain Medicine, Jeonbuk National University Medical School and Hospital, Jeonju 54907, Republic of Korea; gojuno@jbnu.ac.kr (J.H.L.); jth9494@naver.com (T.J.); shko@jbnu.ac.kr (S.K.); 2 Research Institute of Clinical Medicine of Jeonbuk National University-Biomedical Research Institute of Jeonbuk National University Hospital, Jeonju 54907, Republic of Korea

**Keywords:** dexmedetomidine, elderly, obesity, predictors, regional anesthesia, sedation

## Abstract

(1) **Background**: This prospective observational study aimed to investigate the predictors affecting DMT requirements for sedation during regional anesthesia. (2) **Method**: A total of 108 patients who received regional anesthesia with intravenous DMT administration for orthopedic upper- or lower-extremity surgery were enrolled. Following successful regional anesthesia, DMT was administered at a rate of 4 µg/kg/h until reaching loss of consciousness (LOC). The administered dose of DMT per body weight until LOC (DMT_LOC_; µg/kg) was evaluated. The infusion was maintained at a rate of 0.2–0.7 µg/kg/h during the surgery. At the end of surgery, the elapsed time to a BIS value of 90 (T_BIS90_; s) was recorded. Linear regression models were used to identify potential predictors of DMT_LOC_ and T_BIS90_. (3) **Results**: One hundred patients were analyzed. There were negative relationships between DMT_LOC_ and age (r = −0.297, *p* = 0.003) and DMT_LOC_ and body mass index (BMI) (r = −0.425, *p* < 0.001), respectively. Multiple linear regression models revealed that both increasing age and BMI were significantly related to DMT_LOC_ (r^2^ = 0.259, *p* < 0.001), but those variables showed no association with T_BIS90_. (4) **Conclusions**: The results of this study suggest that initial loading of DMT should be carefully titrated to minimize risk in elderly and obese surgical populations.

## 1. Introduction

Dexmedetomidine (DMT), a highly selective α2 adrenergic agonist, is one of the most preferred sedative drugs due to its advantages. It manifests sympatholytic, sedative, hypnotic, amnesic, and analgesic properties. In particular, the combination of intravenous or perineural DMT with regional anesthesia provides notable advantages, including enhanced quality of regional anesthesia [1], prolonged postoperative analgesia [2,3], and opioid-sparing properties [2,3]. Moreover, DMT demonstrates remarkable safety in procedural sedation, with a lower incidence of respiratory depression compared to propofol or midazolam [4].

An initial loading administration of DMT is commonly employed to achieve the desired level of sedation prior to surgical procedures, although it is not recommended for ICU sedation due to the potential risk of hemodynamic instability, such as hypotension or bradycardia [5]. The manufacturer’s recommended initial loading dose of DMT for procedural sedation is 1.0 μg/kg, administered over a period of 10 min. However, it is important to note that this dosage may result in under- or over-sedation in certain populations during clinical practice [6]. Nevertheless, there have been limited investigations regarding the factors that can predict the appropriate initial loading dose of DMT. In particular, identifying the factors associated with a lower DMT requirement is crucial, as it can lead to faster recovery from sedation and promote hemodynamic stability by preventing DMT overdosing.

Obesity can significantly influence the pharmacology of the lipophilic drugs like DMT. Obese patients generally have increased lean body mass as well as fat mass. However, the relative proportion of lean body mass (fat-free mass) to total body weight may decrease, because the increased extent of fat tissue is greater [7]. As a result, the central volume of distribution, which includes plasma and highly perfused tissues, such as brain, liver, and kidney, is relatively less in obese populations compared to those with normal weight [7]. While there is a higher probability of overdosing the lipophilic drug due to the altered pharmacokinetics o the obese population, the clinical effects of obesity on the pharmacology of DMT remain inconsistent according to previous studies [6,8,9].

The purpose of this prospective observational study was to investigate the predictive factors associated with the initial loading dose of DMT required to achieve loss of consciousness (LOC) during regional anesthesia. The hypothesis of the study was that body mass index (BMI) would influence the DMT requirement for achieving LOC.

## 2. Materials and Methods

This prospective, single-arm, non-randomized, interventional study was approved by the Institutional Review Board of the authors’ hospital (IRB number: CUH 2020-12-015) and registered with the WHO International Clinical Trials Registry Platform (KCT0005737). Informed consent was obtained from all participants before enrollment in the study. This manuscript adheres to the applicable STROBE guidelines. One hundred eight patients who were scheduled to undergo orthopedic upper- or lower-extremity surgery under regional anesthesia at our institution were enrolled in this study during the period from January 2021 to June 2023. The inclusion criteria were as follows: aged 18–75 years, American Society of Anesthesiologists physical status (ASA/PS) I-II, and administered DMT for sedation during the surgery. Patients were excluded from the study under the following circumstances: (1) patient refusal, (2) severe hepatorenal impairment, (3) a history of congestive heart failure or depressed left ventricular function (ejection fraction of less than 50%), (4) patients had a pre-anesthetic heart rate of less than 50 beats per minute (bpm), (5) patients had a problem with communication, such as hearing impairment or language difficulties.

### 2.1. Standardized Regional Anesthesia Management

Upon arrival at the operating room, standard anesthesia monitoring, including blood pressure, electrocardiogram, and pulse oximetry, were applied. Additionally, bispectral (BIS) index monitoring was employed to assess the depth of sedation. Each participant received one of the three types of regional anesthesia, which were brachial plexus blocks, spinal anesthesia, or sciatic and femoral nerve blocks, according to the surgical site and the individualized pre-anesthetic plan. The regional anesthesia protocols were standardized for all participants. Under ultrasound guidance, supraclavicular brachial plexus blocks were performed for upper-extremity surgery, while sciatic and femoral nerve blocks were carried out for lower-extremity surgery. A total of 30–35 mL of 1.5% lidocaine with 5 µg/mL epinephrine was injected around the neural sheath during the procedure. In cases involving spinal anesthesia, 10–14 mg of hyperbaric bupivacaine was administered at the L4/5 interspinous space, which was identified using the Tuffier’s line palpation method. The level of the sensory block was carefully adjusted to achieve anesthesia at the T10-L1 region, as determined through a pin-prick test. After confirming the successful motor and sensory block and ensuring hemodynamic stability following regional anesthesia, a DMT infusion was initiated to provide sedation for the patients.

### 2.2. Intervention Protocol and Outcome Assessment

DMT (Precedex^®^, Hospira, Lake Forest, IL, USA, 200 µg in 1 mL) was diluted with 49 mL of 0.9% normal saline at a concentration of 4 µg/mL in a total volume of 50 mL. As an initial loading administration, the solution was infused at a rate of 1 mL/kg/h (equivalent to 4 µg/kg/h of DMT) until the patient reached loss of consciousness (LOC). Throughout the infusion, oxygen was delivered to the patient at 6 L/min via a simple face mask. During this period, the participants were encouraged to firmly hold a baseball with their non-operating hand, palm facing down, and the moment of ball drop was defined as the point of LOC. The administered dose of DMT per patient’s body weight until LOC (DMT_LOC_; µg/kg), the elapsed time to LOC (T_LOC_; min), and BIS value at LOC (BIS_LOC_) were collected.

Subsequently, DMT was infused at a maintenance rate of 0.2–0.7 µg/kg/h during the surgery. The maintenance administration was titrated at 3 min intervals to achieve the level of sedation with target Modified Observer’s Assessment of Alertness/Sedation scale (MOAAS) measurements of 3–4, representing moderate sedation and a BIS value of 60–80. At the end of the surgery, the infusion of DMT was stopped. The cumulative dose of DMT per body weight during surgery (DMT_CUM_; µg/kg) and the elapsed time to a BIS value of 90 (T_BIS90_; min) were recorded. Recovery of consciousness (ROC) was defined as spontaneous eye opening. Vital signs, including noninvasive blood pressure, heart rate, and peripheral oxygen saturation, were continually recorded until the end of anesthesia.

The primary purpose of the study was to investigate the predictive factors associated with DMT_LOC_. Linear regression analysis was utilized to determine the predictors for DMT_LOC_. The statistically significant variables proved by univariate analysis were then integrated into a multivariate linear regression model.

### 2.3. Sample Size Calculation and Statistical Analysis

The sample size of the current study was predetermined by the correlation sample size calculation using SigmaPlot 14.5 (Systat Software Inc., San Jose, CA, USA) based on the assumption that the correlation coefficient between BMI and DMT_LOC_ would be 0.3. A total of 85 patients were required with a significance level of 0.05 (α = 0.05) and power of 80% (β = 0.20). To allow for attrition, the total sample size was increased to 110.

Statistical analysis was conducted using SigmaPlot 14.5. Continuous and categorical variables were analyzed using two-tailed independent-sample *t*-tests and Mann–Whitney rank-sum tests, respectively, after assessing normality. For comparisons involving three or more groups, the one-way analysis of variance (ANOVA) or ANOVA on ranks was employed following normality test. Before multivariate analysis, univariate correlation analysis was performed between the independent and dependent variables. And linear regression analysis was utilized to determine the predictors for DMT_LOC_. The statistically significant variables proved by univariate analysis were then integrated into a multivariate linear regression model, and regression coefficient (β), coefficient of determination (R^2^), and *p*-values were calculated for each variable. Continuous hemodynamic parameters, including blood pressure and heart rate, along with BIS value, were analyzed by one-way ANOVA. All descriptive data are expressed as mean ± standard deviations (SDs), median [interquartile range], and number of patients (%). Differences with a *p*-value < 0.05 were considered statistically significant.

## 3. Results

### 3.1. Study Participants and Patient Characteristics

**Figure 1:**
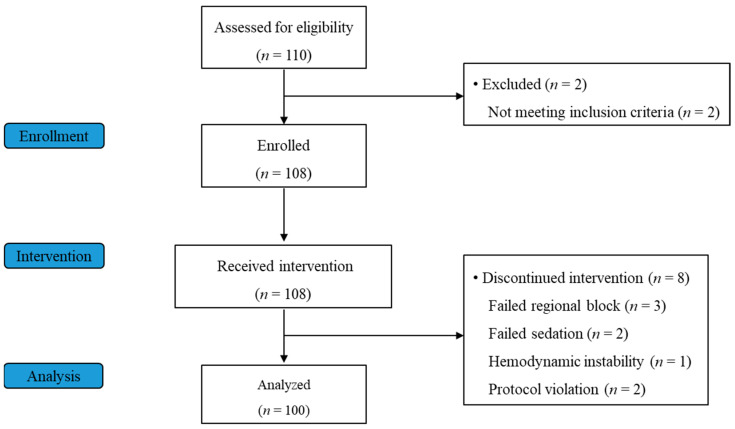
Subject flow diagram.

Among the 108 participants enrolled, 100 subjects who completed the intervention were analyzed in the current study. The success rate for regional anesthesia was 97.2%. The patient flow chart is shown in Figure 1. Among the eight patients who did not complete the study, two patients were excluded because they failed to reach LOC despite increasing the initial loading dose of DMT to over 1.5 µg/kg (failed sedation). One patient experienced hemodynamic instability due to the high spinal block above T5, and protocol violation included machine and measurement errors during the intervention.

The patients’ demographics are presented in Table 1. The mean age of the participants was 46.3 ± 17.6 years with the minimum (Min) and maximum (Max) being 18 and 74 years, respectively. And the patients’ BMIs (kg/m^2^) were distributed from 19.24 (Min) to 35.7 (Max) with a median value of 25.4. Among the 100 participants included in the study, 54 of them received brachial plexus blocks for upper-extremity surgery, and 43 participants underwent lower-extremity surgery under spinal anesthesia. In a smaller subset of cases, specifically, for three participants, femoral/sciatic nerve blocks were administered.

### 3.2. Study Outcomes

Regarding DMT sedation characteristics (shown in Table 2), the median DMT_LOC_ values (µg/kg) were 0.83 and 0.89 for men and women, respectively (*p* = 0.303), and T_LOC_ (min) was also comparable between men and women. The median BIS_LOC_ values were 82.0 and 81.0 for men and women, respectively (*p* = 0.249). In the perspective of recovery profiles, the T_BIS90_ (min) values were 4.6 (2.9–9.6) and 5.0 (2.3–9.8) minutes for men and women, respectively (*p* = 0.911).

**Figure 2:**
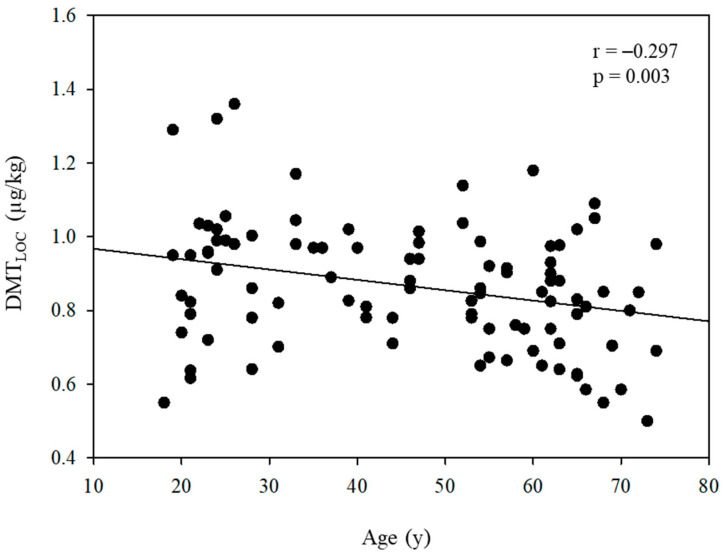
Linear regression analysis between the administered dose of dexmedetomidine for loss of consciousness (DMT_LOC_) and age.

**Figure 3:**
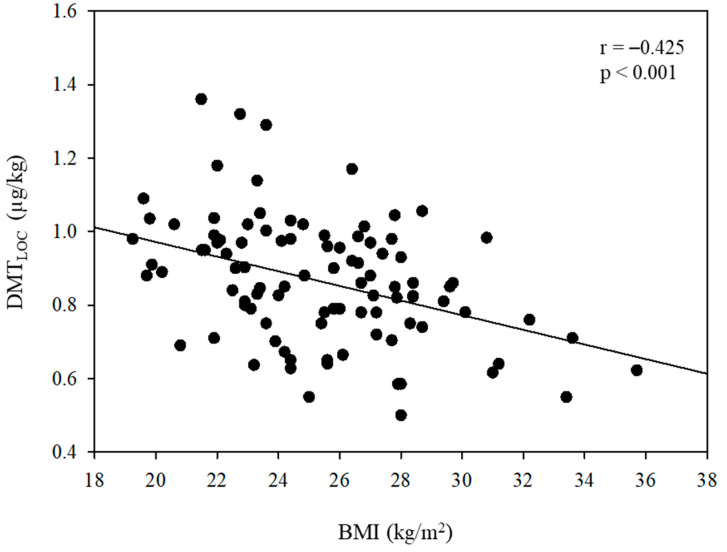
Linear regression analysis between the administered dose of dexmedetomidine for loss of consciousness (DMT_LOC_) and body mass index (BMI).

In Figure 2, a negative correlation between DMT_LOC_ and age is depicted, with a correlation coefficient (r) of −0.297 (*p* = 0.003). Figure 3 further demonstrates a negative relationship between DMT_LOC_ and BMI (r = −0.425, *p* < 0.001).

The results of the univariate and multivariate linear regression analyses against DMT_LOC_ are shown in Table 3.

The formula obtained using multiple linear regression analysis was as follows:

DMT_LOC_ (µg/kg) = 1.590 − (0.030 × “age” (“every 10 years”) − (0.023 × BMI) (R^2^ = 0.259, *p* < 0.001)

In the recovery profile, there were no significant correlations between the patient- and DMT-related variables, including age, BMI, sex, DMT_CUM_ (µg/kg), and T_BIS90_ (Table 4).

We performed further analysis with stratified patient groups according to BMI (18.5–24.9 kg/m^2^: normal weight, 25.0–29.9 kg/m^2^: overweight, ≥30.0 kg/m^2^: obese). A summary of the comparisons of the patient and DMT sedation characteristics is presented in Table 5. There was a significant difference between the DMT_LOC_ (µg/kg) values of the normal-weight, overweight, and obese groups according to one-way ANOVA on ranks with multiple comparisons (0.94 vs. 0.83 vs. 0.68, *p* < 0.001). Both DMT_LOC_ and T_LOC_ were significantly lower in the obese compared to normal-weight groups, respectively, according to a Dunn’s post hoc test (*p* < 0.001 and *p* = 0.009). T_BIS90_ was comparable among the normal-weight, overweight, and obese groups.

**Figure 4:**
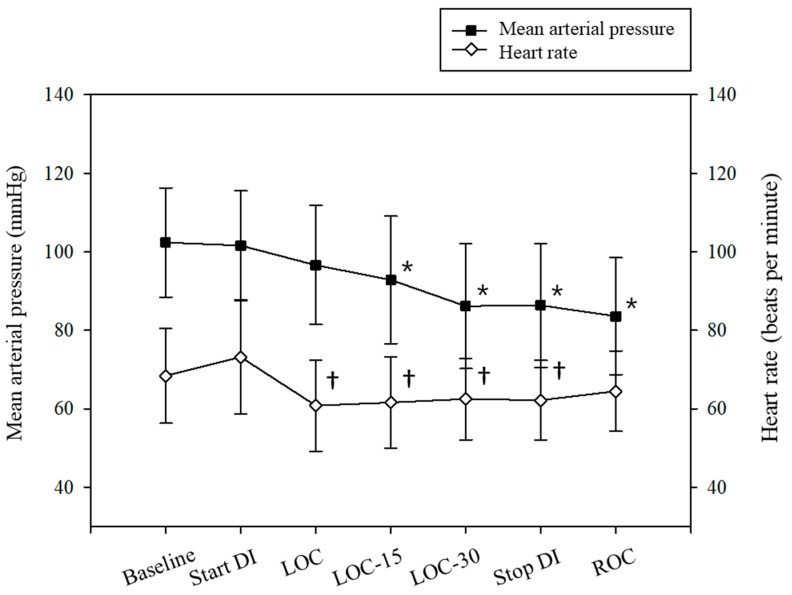
Hemodynamic profile, including mean arterial pressure and heart rate. DI, dexmedetomidine infusion; LOC, loss of consciousness; LOC-15, 15 min following LOC; LOC-30, 30 min following LOC; ROC, recovery of consciousness. * *p* < 0.05 compared to baseline, † *p* < 0.05 compared to baseline.

**Figure 5:**
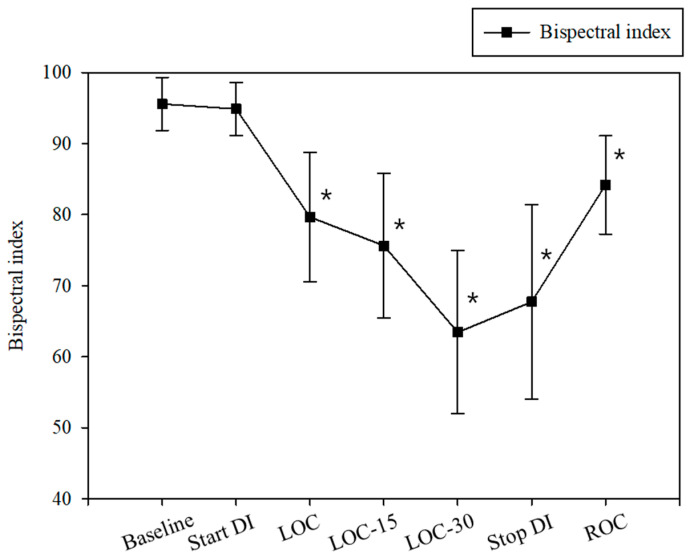
Bispectral index. DI, dexmedetomidine infusion; LOC, loss of consciousness; LOC-15, 15 min following LOC; LOC-30, 30 min following LOC; ROC, recovery of consciousness. * *p* < 0.05 compared to baseline.

The hemodynamic profile, including mean arterial pressure and heart rate and BIS values during the intervention, are presented in Figure 4 and Figure 5.

## 4. Discussion

The current study examined the characteristics of DMT-induced sedation during extremity surgery under regional anesthesia. This study found that the average initial DMT loading requirements were 0.83 µg/kg and 0.89 µg/kg for men and women, respectively. These values are consistent with a previous study that reported that a 95% effective dose (ED95) of DMT was 0.86 µg/kg [10]. These values are notably lower than the commonly used regimen of 1.0 µg/kg. And the BIS values at LOC (defined as the moment of ball drop) were 82.0 and 81.0 for men and women. We also aimed to examine the predictive factors that influence the initial DMT loading requirements. The primary finding suggests that the initial loading dose of DMT should be reduced for older or obese patients. In the perspective of recovery from sedation, our study found that patients consistently experienced a rapid recovery from sedation within 10 min, irrespective of their demographics, after a continuous infusion of DMT for approximately 1 h.

Indeed, the aging process has a substantial impact on the pharmacology of various sedative drugs, which is primarily attributable to alterations in pharmacokinetics and pharmacodynamics. Notably, older patients generally have a reduced drug metabolism and clearance as well as an increased sensitivity to the sedatives. Such age-related changes carry significant clinical implications that require careful consideration in clinical practice. However, the impact of age on the pharmacology of DMT remains in conflict based on previous studies. Iirola et al. suggested that increasing age and hypoalbuminemia might influence the pharmacokinetics of DMT, such as through decreased drug clearance [11]. In contrast, other pharmacokinetic studies of DMT have shown that age dose not significantly affect its pharmacokinetic profiles [12,13]. Meanwhile, it has been observed that the sedative effect of DMT could be more profound in older patients compared to young adults, indicating an increased sensitivity to the drug [14]. The current study also found a negative correlation between increasing age and the initial DMT loading requirement (r = −0.297, *p* = 0.003). Based on these results, the authors propose considering age-based adjustments to the initial loading dose of DMT. This approach aims to optimize sedation while minimizing the risk of adverse effects that may arise from excessive administration.

Several studies have indicated that the pharmacology of DMT could be affected in obese patients, although the clinical effects of obesity on the pharmacology of DMT remain inconclusive [6,8,9]. For instance, Xu et al. reported that morbidly obese (BMI > 40 kg/m^2^) patients exhibited a deeper level of sedation with a higher peak plasma concentration of DMT compared to normal-weighted individuals when given a dose of 1 µg/kg of DMT [6]. In line with previous findings, the current study demonstrated a significant reduction of 11.7% in the initial DMT loading requirement for the overweight population and one of 27.7% for the obese population when compared to normal-weight individuals. These findings indicate that adjusting dosage based on BMI may be relevant not only for the morbidly obese population, as discussed in previous studies, but also for those who are overweight. Furthermore, our previous study revealed a significant association between obesity and the occurrence of DMT-induced hypotension when DMT was administered based on total body weight [15]. Hence, it is evident that applying an individualized dosing scheme, rather than conventional total-body-weight-based dosing, is crucial for administering DMT to obese populations. This approach is essential to ensure optimal anesthesia and minimize hemodynamic risks in this specific patient group.

The authors hypothesized that the recovery from sedation following continuous infusion of DMT might be slower for obese populations compared to normal-weight individuals. The pharmacokinetics of DMT are known to follow a two-compartment model [11,16], and DMT can be potentially accumulated in a relatively larger amount of body fat, which comprises peripheral compartments. Additionally, the clearance of DMT, which is primarily dependent on hepatic blood flow, may be reduced in the morbidly obese population, although the exact mechanism remains unclear [6]. However, in the current study, there was no significant difference between the DMT recovery profiles of the obese and normal-weight groups (Table 4). The initial loading and maintenance doses of DMT were adjusted to maintain an appropriate depth of sedation, and the DMT_CUM_ (µg/kg) was decreased in the obese group. Although DMT can continuously release from peripheral compartments, such as from adipose tissue into plasma, the hepatic metabolism of DMT can rapidly decrease plasma concentrations to the arousal level within a few minutes in clinical anesthesia practice [13]. Therefore, the recovery from sedation was not found to be influenced by obesity in the current study.

The current study has some limitations. Firstly, plasma concentrations of DMT were not measured in order to demonstrate the pharmacological effects. The similar or higher plasma concentrations of DMT in older or obese patients following an initial loading administration may suggest potential pharmacokinetic changes, including alterations in the drug distribution of DMT. Further investigations combining pharmacokinetic and pharmacodynamic studies would be valuable in providing a clearer understanding of the pharmacologic alterations in these specific patient populations. Secondly, inter-individual variability was not considered in this study. Several patient-related factors, including hypoalbuminemia and lower cardiac output, were suggested to be associated with prolonged effects of DMT in critically ill patients [11]. DMT has a high protein-binding capability, binding at 94% to albumin and α1-glycoprotein. Therefore, hypoalbuminemia can be expected to affect the pharmacokinetics of the drug [11,13]. However, it is worth mentioning that all participants enrolled in this study were healthy patients with ASA/PS I or II, and individuals with serious medical conditions were excluded from the study. Thirdly, examining recovery from sedation after discontinuing DMT infusion was complicated. DMT produces a unique sedative appearance, known as “cooperative” or “arousable” sedation, where patients can easily awaken from sedation with verbal command or light tactile stimuli. As a result, the assessment of recovery from sedation using conventional methods such as the “open your eyes” response is significantly limited. Fourthly, there was no control group, and the regional anesthesia techniques were not similar, therefore adding to heterogeneity of the study population.

## 5. Conclusions

In conclusion, both increasing age and BMI are independent predictors for reducing the DMT requirement to achieve LOC. These results emphasize that the initial loading dose of DMT should be carefully titrated to minimize potential risks for older and obese surgical populations.

## Figures and Tables

**Table 1:** Patient demographics.

	Men	Women	Total
Number of patients	49	51	100
Age (years)	41.3 ± 16.2	51.0 ± 17.7	46.3 ± 17.6
Height (cm)	172.1 ± 7.1	157.2 ± 6.1	164.4 ± 9.9
Body weight (kg)	78.5 ± 9.9	58.0 ± 7.8	68.9 ± 12.9
BMI (kg/m^2^)	26.7 (23.9–28.4)	23.6 (21.9–26.1)	25.4 (22.9–27.7)
ASA/PS 1/2	11/38	10/41	21/79
Underlying disease [*n* (%)]			
Hypertension	8 (16.3%)	12 (23.5%)	20 (20.0%)
Diabetes mellitus	4 (8.2%)	2 (3.9%)	6 (6.0%)
Type of anesthesia [*n* (%)]			
Brachial plexus block	25 (51.0%)	29 (56.9%)	54 (54.0%)
Spinal anesthesia	23 (46.9%)	20 (39.2%)	43 (43.0%)
Femoral/sciatic block	1 (2.0%)	2 (3.9%)	3 (3.0%)
Surgery time (min)	47.0 (30.5–67.8)	39.0 (30.0–65.0)	44.0 (30.0–66.0)
Anesthesia time (min)	77.5 (60.0–100.0)	72.0 (60.0–100.0)	75.0 (60.0–100.0)
Total infused fluid volume (mL)	251.9 ± 135.0	217.6 ± 121.2	234.2 ± 128.6

Data are presented as number, mean ± SDs, median (interquartile range), or number (%). BMI, body mass index; ASA/PS, American Society of Anesthesiologists physical status.

**Table 2:** Dexmedetomidine sedation characteristics.

	Men (*n* = 49)	Women (*n* = 51)	*p*-Value
The administered dose of DMT for LOC (μg/kg) *	0.83 (0.75–0.97)	0.89 (0.75–1.02)	0.303
The elapsed time to LOC (min) ^†^	12.0 (10.1–13.6)	12.8 (10.5–14.6)	0.449
BIS value at LOC	82.0 (77.0–88.0)	81.0 (72.0–86.0)	0.249
BIS value at the end of surgery ^‡^	69.7 ± 12.1	65.9 ± 15.0	0.162
Cumulative dose of DMT (μg/kg) during surgery	1.1 (0.9–1.3)	1.2 (1.0–1.3)	0.526
The elapsed time to BIS 90 (min) ^§^	4.6 (2.9–9.6)	5.0 (2.3–9.8)	0.911

Data are presented as number and mean ± SDs and median (interquartile range). DMT, dexmedetomidine; LOC, loss of consciousness; BIS, bispectral index. * The initial loading dose of dexmedetomidine until loss of consciousness when infused at a rate of 4 µg/kg/h. ^†^ The elapsed time to loss of consciousness when dexmedetomidine was infused at a rate of 4 µg/kg/h. ^‡^ At the end of the surgery, dexmedetomidine infusion was stopped. ^§^ The recovery time to bispectral index of 90 from stopping dexmedetomidine infusion.

**Table 3:** Regression coefficient (β) and coefficient of determination (R^2^) against the administered dose of dexmedetomidine per body weight for loss of consciousness (DMT_LOC_).

Variable	Univariate Analysis				Multivariate Analysis			
	β	SE	R^2^	*p*-Value	β	SE	R^2^	*p*-Value
							0.259	<0.001 ^†^
Age, 10 yr	−0.032	0.010	0.088	0.003 *	−0.030	0.010		0.002
BMI (kg/m^2^)	−0.020	0.005	0.181	<0.001 *	−0.023	0.005		<0.001 ^†^
Female	0.041	0.038	0.012	0.280				
Anesthesia type (spinal anesthesia)	−0.060	0.038	0.028	0.098				

BMI, body mass index. * *p* < 0.05 by univariate regression analysis. ^†^ *p* < 0.05 by multivariate regression analysis.

**Table 4:** Regression coefficient (β) and coefficient of determination (R^2^) against the elapsed time to BIS value of 90 (T_BIS90_).

Variable	Univariate Analysis			
	β	SE	R^2^	*p*-Value
Age, 10 year	0.412	0.540	0.006	0.447
BMI (kg/m^2^)	0.103	0.282	0.001	0.716
Female	−0.515	1.894	0.001	0.786
DMT_CUM_ (µg/kg)	−5.244	3.146	0.028	0.099

**Table 5:** Comparisons of patient- and dexmedetomidine sedation characteristics according to the body mass index.

	Normal Weight (*n* = 48)	Overweight (*n* = 43)	Obese (*n* = 9)	*p*-Value
Age (years)	49.5 (24.5–63.0)	53.0 (31.0–62.0)	45.5 (26.0–61.8)	0.840
Female sex [*n* (%)]	31 (64.6%)	17 (39.5%)	3 (33.3%)	0.032 *
The administered dose of DMT for LOC (μg/kg)	0.94 (0.81–1.00)	0.83 (0.75–0.94)	0.68 (0.62–0.78)	<0.001 ^†^
The elapsed time to LOC (min)	13.0 (11.1–14.4)	11.7 (10.2–13.6)	9.0 (8.9–11.9)	0.009 ^‡^
BIS value at LOC	82.0 (74.0–87.8)	81.0 (73.0–85.0)	78.5 (65.5–84.5)	0.295
BIS value at the end of surgery	64.6 ± 13.2	71.5 ± 13.9	65.9 ± 12.8	0.051
Cumulative dose of DMT (μg/kg)	1.2 (1.0–1.3)	1.1 (0.9–1.3)	0.9 (0.7–1.1)	0.006 ^§^
The elapsed time to BIS 90 (min)	5.0 (2.9–9.8)	4.8 (2.6–10.5)	3.2 (2.3–4.9)	0.460

Data are expressed as mean ± SD or median (interquartile range). DMT, dexmedetomidine; LOC, loss of consciousness; BIS, bispectral index. Normal weight: a body mass index of 18.5–24.9 kg/m^2^, overweight: 25.0–29.9 kg/m^2^, and obese: ≥30.0 kg/m^2^. * There were no significant differences based on multiple comparisons with Dunn’s post hoc test. ^†^ *p* < 0.05 between normal-weight and overweight groups, as well as between normal-weight and obese groups, according to multiple comparisons using Dunn’s post hoc test. ^‡^ *p* < 0.05 between normal-weight and obese groups according to multiple comparisons. ^§^ *p* < 0.05 between normal-weight and overweight groups, as well as between overweight and obese groups, according to multiple comparisons.

## Data Availability

The raw data supporting the conclusions of this article will be made available by the authors on request.
